# Reversible Cerebral Vasoconstriction Syndrome Following a Steroid Burst: A Case Report

**DOI:** 10.5811/cpcem.53083

**Published:** 2026-04-07

**Authors:** Jacob Lenning, Caleb Halfill, Justin Rountree

**Affiliations:** Western Michigan University Homer Stryker M.D. School of Medicine, Department of Emergency Medicine, Kalamazoo, Michigan

**Keywords:** reversible cerebral vasoconstriction syndrome, thunderclap headache, glucocorticoids, case report

## Abstract

**Introduction:**

Reversible cerebral vasoconstriction syndrome is a recently defined disease entity classically presenting with recurrent thunderclap headache. The pathology involves triggered cerebral arterial vasoconstriction, which can lead to complications including seizure, ischemic stroke, and intracranial hemorrhage. Diagnosis requires angiography, and treatment consists of vasodilatory therapy.

**Case Report:**

We describe a case of reversible cerebral vasoconstriction syndrome following glucocorticoid burst therapy in a patient on multiple vasoactive medications, suggesting the possibility of compounding risk factors and triggers. As is common with this syndrome, the patient in our case required multiple hospital visits for diagnosis but ultimately experienced a positive outcome upon treatment.

**Conclusion:**

The presentation of reversible cerebral vasoconstriction syndrome can vary. Diagnosis requires a high degree of suspicion in patients with potential triggers to ensure early treatment and avoidance of poor outcomes.

## INTRODUCTION

Reversible cerebral vasoconstriction syndrome is an under-recognized cause of headache often requiring multiple medical encounters before diagnosis.^1^ Recurrent thunderclap headache—a sudden headache with maximal intensity upon onset—is the classic chief complaint; associated features such as focal neurological deficits are not uncommon.^2^,^3^ The pathophysiology involves triggered cerebral arterial vasoconstriction.^4^,^5^ Reversible cerebral vasoconstriction syndrome is often diagnosed on computed tomography angiography (CTA) or magnetic resonance angiography (MRA) after other causes of thunderclap headache such as subarachnoid hemorrhage are ruled out. The gold standard for diagnosis is cerebral digital subtraction angiography.^6^,^7^

Most patients with reversible cerebral vasoconstriction syndrome have an excellent prognosis. However, sequelae may include cerebral infarction and hemorrhage leading to increased morbidity and mortality.^6^,^7^ Therefore, awareness of this syndrome and potential triggers is imperative. We present the second reported case of reversible cerebral vasoconstriction syndrome following glucocorticoid therapy. The first reported case was thought to be related to steroid burst therapy.^8^ In the case we describe here the temporal relationship alone does not prove glucocorticoid therapy was the trigger. While studies have demonstrated worse outcomes when glucocorticoids were used in the treatment of the syndrome, none have confirmed a triggering effect. Further investigation is needed.^7^ However, this case does prompt consideration of a compounding risk factor and trigger effect.

## CASE REPORT

A 49-year-old female with a past medical history including Crohn disease, hypertension, and insulin-dependent diabetes, prescribed venlafaxine for mood disorder and estradiol for vasomotor menopausal symptoms, presented to the emergency department (ED) with four days of intermittent headaches. The patient described each episode as a sudden “boom” with severe intensity. Review of the medical record revealed that she had completed a three-day burst of methylprednisolone 1 gram daily prior to symptom onset and was continued on a prednisone taper for pyoderma gangrenosum of the lower extremity. Her initial blood pressure was 179/77 millimeters of mercury with otherwise normal vital signs. She was lying on the stretcher with the lights off and appeared uncomfortable. The physical examination revealed no focal neurological deficits, and the lower extremity wound appeared to be non-infected.

**Image 1:**
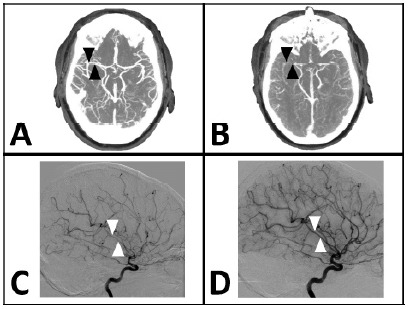
**(A)** Computed tomography angiography (CTA) of the head upon initial presentation demonstrating the normal-appearing middle cerebral artery (black arrows) compared to the repeat CTA head**; (B)** demonstrating segmental vasoconstriction (black arrows); **(C)** cerebral digital subtraction angiography (DSA) demonstrating segmental vasoconstriction (white arrows) compared to DSA immediately following intra-arterial verapamil; and **(D)** resolution of the vasoconstriction (white arrows).

Subarachnoid hemorrhage was considered to be the most likely diagnosis. Computed tomography brain without contrast revealed no acute intracranial hemorrhage and was grossly normal. The CTA head and neck revealed a possible 2-mm aneurysm arising from the right posterior cerebral artery (not imaged) without evidence of rupture but was otherwise normal (Image 1A). Despite diphenhydramine, metoclopramide, and acetaminophen, the headache remained unchanged. Therefore, the neurointerventionalist was consulted and took the patient for diagnostic cerebral digital subtraction angiography, which demonstrated no actual aneurysm. Further inpatient workup included a lumbar puncture with normal opening pressure and unremarkable cerebrospinal fluid analysis. The headaches improved with blood pressure control, and the patient was discharged home.

Two days later, she was brought back to the ED by the family for confusion. The family stated the patient was “slow to respond” during conversation and was no longer independent with her activities of daily living. The patient endorsed the return of her headache as well. She was alert and oriented without focal neurological deficits but was clearly slow in her verbal responses and had difficulty providing history.

Repeat CT brain without contrast identified acute infarctions in the right cerebellum and left parietal lobe. Upon admission, repeat CTA head and neck revealed extensive vasoconstriction of the intracranial vasculature with diminished flow through the cerebral arteries compared to the previous study (Image 1B). Differential diagnosis included acute vasculitis and reversible cerebral vasoconstriction syndrome. The neurointerventionalist performed a repeat cerebral digital subtraction angiography demonstrating the classical segmental vasoconstriction of the syndrome (Image 1C). An intra-arterial infusion of verapamil was administered, resulting in the reversal of vasoconstriction and confirming the diagnosis of reversible cerebral vasoconstriction syndrome (Image 1D).

The following day, the patient reported that her headache had completely resolved. She was placed on extended-release verapamil by mouth daily, and she was switched from venlafaxine to lamotrigine for her mood disorder. Additionally, she was taken off estrogen, although neurology consult felt the final few low-doses of the oral prednisone taper could be continued for the pyoderma gangrenosum. The patient was discharged two days after the therapeutic intra-arterial verapamil infusion. Upon follow-up a few months later, she was headache-free and back to her normal daily activities.

*CPC-EM Capsule*

What do we already know about this clinical entity?

*Reversible cerebral vasoconstriction syndrome is a recently defined disease entity classically presenting with recurrent thunderclap headache*.

What makes this presentation of disease reportable?

*This case of a patient who developed the syndrome following glucocorticoid burst therapy highlights multiple possible risk factors and triggers*.

What is the major learning point?

*Diagnosis requires a high degree of suspicion in patients with potential triggers to ensure early treatment and avoidance of poor outcomes*.

How might this improve emergency medicine practice?

*Increased awareness of reversible cerebral vasoconstriction syndrome as a possible cause of thunderclap headache may hasten diagnosis and treatment*.

## DISCUSSION

As in the case presented here, reversible cerebral vasoconstriction syndrome most commonly presents with thunderclap headache in female patients 40–60 years of age,^2^,^3^,^9^ although the syndrome has been recognized in men and children as well.^3^ In addition to female sex, use of vasoactive medications is one of the most commonly reported triggers.^3^,^5^ Others include blood transfusions, immunosuppressants, surgery, trauma, catecholamine secreting tumors, autonomic dysfunction, recent pregnancy, arterial disorders, arterial dissections, systemic lupus erythematosus, migraine headaches, and even lifestyle changes.^5^,^6^,^9^,^10^

The patient in our case had multiple possible triggers for the syndrome. She was taking venlafaxine and estrogen therapy, both vasoactive medications. She had also just completed a steroid burst, and although no studies provide direct evidence of glucocorticoids triggering the syndrome, one prior case report describes a similar temporal relationship..Steroids are known to worsen the condition^7^,^8^; therefore, even if not the trigger, it is possible the steroid burst therapy exacerbated the disease process in this patient. Further study is necessary to determine whether glucocorticoids trigger reversible cerebral vasoconstriction syndrome. The multiple potential triggers in this case raise the possibility of a compounding risk factor and trigger effect, although this hypothesis would require further investigation.

The exact pathophysiology of reversible cerebral vasoconstriction syndrome is unknown; there are multiple proposed mechanisms including disturbances in cerebral vascular tone, sympathetic overactivity, endothelial dysfunction, oxidative stress, and blood-brain barrier breakdown.^5^,^11^ Previous studies report no histologic or inflammatory changes in brain or arterial biopsies of patients diagnosed with the syndrome and, therefore, biopsy is not routinely recommended if it is suspected.^4^ Catecholamines, endothelin-1, serotonin, nitric acid, and prostaglandins are associated with vasospasm in aneurysmal subarachnoid hemorrhage, and these factors may have similar role in reversible cerebral vasoconstriction syndrome, although further investigation is needed.^11^ Posterior reversible encephalopathy syndrome shares many features with reversible cerebral vasoconstriction syndrome, Some authors hypothesize the entities to reside on the same spectrum of disease.^4^ The current expert consensus considers it to be the endpoint of multiple disease states, which is reflected in previous terms for the syndrome including migrainous vasospasm, Call-Fleming syndrome, and benign and idiopathic thunderclap headache.^1^,^2^,^4^

Thunderclap headache is the predominant presenting feature of reversible cerebral vasoconstriction syndrome. Patients may experience multiple, recurrent thunderclap headaches, although this historical feature is not completely sensitive nor is it specific for diagnosis of the syndrome.^3^,^5^,^9^ One study estimated that up to 8.8% of thunderclap headaches presenting to the ED are due to reversible cerebral vasoconstriction syndrome, although actual incidence may be higher due to overall low awareness among clinicians.^12^ Photophobia, vomiting, seizures, focal neurological deficits, vision changes, encephalopathy, and acute hypertension are other possible presenting features of the syndrome but are even less sensitive and specific.^4^,^5^,^11^ Physical exams are typically non-focal but may occasionally demonstrate neurological deficits. Generally, the syndrome is characterized by variable presentations making recognition difficult for clinicians.^9^,^11^

The differential diagnosis for reversible cerebral vasoconstriction syndrome includes other causes of thunderclap headaches such as subarachnoid hemorrhage, intracerebral hemorrhage, cerebral venous sinus thrombosis, cervical artery dissection, cerebral infarctions, central nervous system (CNS) tumors, and CNS infections.^5^ Bloodwork and cerebrospinal fluid analysis can evaluate for these other causes but will not directly make the diagnosis of this syndrome. As in our case, symptoms often precede identifiable radiological changes. Initial CT and magnetic resonance imaging are initially normal in many patients: 55% in one study, with most patients (81%) demonstrating abnormal findings upon repeat imaging.^3^ Radiographic studies may exhibit subarachnoid hemorrhage, intracranial hemorrhage, and cerebral infarction. Cerebral edema with mass effect and herniations are less commonly seen.^11^,^13^ Cerebral infarctions in reversible cerebral vasoconstriction syndrome often appear bilaterally, especially in watershed regions. Intracerebral hemorrhage tends to be multifocal as well.^11^,^13^

Cerebral digital subtraction angiography is the gold standard diagnostic tool with 100% sensitivity for the typical cerebral vasoconstrictive pattern of the syndrome, if present. Less invasive CTA and MRA studies are around 80% sensitive.^6^ The typical angiography findings include alternating areas of vasoconstriction and dilation forming a “sausage on a string” or “string of beads” pattern as seen in this case (Image 1B, C).^6^,^10^,^11^ Diagnostic confirmation of reversible cerebral vasoconstriction syndrome requires observed reversibility of vasospasm on imaging.^11^ To aid in the diagnosis, a few scoring tools have been developed, but none have been validated.^6^ Some studies suggest that progression of the syndrome can be monitored with transcranial Doppler ultrasound by evaluating maximum mean flow velocity in the middle cerebral artery, although diagnostic value is limited with sensitivity of 42–67%.^7^

Urgent recognition of reversible cerebral vasoconstriction syndrome is necessary to withdraw triggers and limit severe effects of complications that can be associated with significant morbidity and even mortality.^1^,^5^ Life-threatening conditions such as seizure, ischemic stroke, and intracranial hemorrhage can be caused by cerebral vasoconstriction associated with the syndrome.^1^,^2^ The reported complication rates vary, with up to 43% of patients experiencing intracranial hemorrhage, 33% ischemic stroke, and 28% posterior reversible encephalopathy syndrome.^6^ Morality rates up to 6.8% have been reported.^6^

Treatment of reversible cerebral vasoconstriction syndrome is largely supportive and includes symptom control, management of complications, withdrawal of triggers, and initiating vasodilator therapy with calcium channel blockers and magnesium sulfate.^5^,^6^ First-line treatment with calcium channel blockers such as nimodipine have been shown to improve headache intensity, although they have not been proven to alter duration or mitigate the risk of complications.^5^,^6^,^13^ Treatment with calcium channel blockers must be done carefully to avoid associated hypotension, which may increase the risk of infarction in vascular watershed areas.^10^

Additional therapies include intra-arterial treatment with vasodilators and balloon angiography, which improve artery caliber; however, no studies have shown clear clinical benefit.^5^,^6^ Treatment with high-dose glucocorticoids was historically common; however, multiple studies have described poor outcomes with steroid treatment.^5^–^7^,^11^ No studies report evidence of reversible cerebral vasoconstriction syndrome triggered by glucocorticoids, although the temporal relationship in our case and the one prior case report suggests further investigation is warranted.^8^ Ultimately, most patients diagnosed with the syndrome will have an excellent prognosis following supportive care and removal of triggers, making awareness of it and the potential triggers imperative.

## CONCLUSION

Reversible cerebral vasoconstriction syndrome is a recently defined disease entity and should be routinely included in the differential diagnosis for thunderclap headache. The presentation is often variable, requiring emergency clinicians to maintain high suspicion in patients with possible triggers, especially if multiple risk factors and potential triggers are present. Early identification will ensure patients receive timely care with management of any complications to limit morbidity and mortality.
